# Why is congenital Zika syndrome asymmetrically distributed among human populations?

**DOI:** 10.1371/journal.pbio.2006592

**Published:** 2018-08-24

**Authors:** Jimena Barbeito-Andrés, Lavínia Schuler-Faccini, Patricia Pestana Garcez

**Affiliations:** 1 Institute of Biomedical Sciences, Federal University of Rio de Janeiro, Rio de Janeiro, Brazil; 2 Institute for Studies in Neuroscience and Complex Systems Studies, ENyS, CONICET, Buenos Aires, Argentina; 3 Departament of Genetics, Institute of Biosciences, Universidade Federal do Rio Grande do Sul, Porto Alegre, Brazil; 4 Instituto Nacional de Genetica Medica Populacional, Medical Genetics Service, Hospital de Clinicas de Porto Alegre, Porto Alegre, Brazil; Imperial College London, United Kingdom of Great Britain and Northern Ireland

## Abstract

Zika virus (ZIKV) is a health burden due to the severe neurological abnormalities that arise after congenital infection. Although multiple experimental studies have linked ZIKV with neural birth defects, the scientific community has not been able to fully explain why Congenital Zika Syndrome (CZS) was only apparent after the virus entered the Americas and why these occurrences have an asymmetric geographic distribution. Here, we review the impact of ZIKV infection on human populations by exploring evolutionary changes in the virus’ genome as well as examining the diverse genetic and environmental cofactors of the human hosts.

## Background

Although Zika virus (ZIKV) was first isolated in 1947 in Uganda [1] and has been circulating in human populations along West Africa and Asia since the mid-1950s, little attention was devoted to it until the beginning of the 21st century [2–4]. Probably, the mildness of its symptoms and the fact that it was geographically restricted resulted in ZIKV being a neglected disease for most of its history. However, this started to change in 2013 when the landing of ZIKV in French Polynesia was associated with increased rates of Guillain–Barre syndrome, an autoimmune condition that affects the peripheral nervous system and can be triggered by infections. In 2015, only two years after the Polynesian outbreak, an unprecedented epidemic was registered in Brazil. There, ZIKV became an extraordinary health burden due to the novel correlation between it and severe brain malformations in newborns. Rapidly, several studies endorsed this causal relationship—between ZIKV and brain development disorders [5–7]—with some authors stating that there was enough evidence to consider it a new member of the teratogenic congenital infections of the Syphilis, Toxoplasmosis, others, Rubella, Cytomegalovirus, and Herpes virus (STORCH) group [8]. As cases accumulated, the term Congenital Zika Syndrome (CZS) was proposed, which stands for a spectrum of different neuropathological conditions that emerge after ZIKV intrauterine transmission [9–11] (Table 1). Despite a notable increase in scientific focus, which reached feverish heights after a World Health Organization emergency alert, the path of CZS leaves some puzzling questions unsolved.

**Table 1:** Features commonly described in cases of CZS newborns.

Type of abnormality	Description
Microcephaly	Reduction of head (skull and brain) size. Usually, cases in which head circumference is at least two standard deviations smaller than the gestational age and body size average are considered microcephalic. In CZS cases, a collapsed skull with overlapping sutures, preeminent occiput, and marked craniofacial disproportion are observed.
Calcifications	Abnormal calcium deposits. These can be diagnosed via computed tomography or magnetic resonance imaging. In CZS, subcortical calcifications are usually observed, while in other STORCH infections, periventricular calcifications are more common.
Ventriculomegaly	Abnormal dilatation of lateral ventricles.
Lissencephaly	Cortical surface is abnormally smoothed. It is usually accompanied by defects of gyrification.
Corpus callosum abnormalities	Hypoplasia, dysgenesis, and agenesis of the corpus callosum are reported.
Eye abnormalities	Macular scarring and focal pigment retinal mottling are some of the most frequent characteristics.
Other disorders	Hypertonia, extrapyramidal signs, and congenital contractures in the limbs.

**Abbreviations:** CZS, Congenital Zika Syndrome; STORCH, Syphilis, Toxoplasmosis, others, Rubella, Cytomegalovirus, and Herpes virus.

ZIKV is an RNA virus from the Flaviviridae family, which has an 11-kb genome encoding seven nonstructural (NS1, NS2A, NS2B, NS3, NS4A, NS4B, NS5) and three structural proteins (C, prM, and E) [12]. From a phylogenetic point of view, there are two main lineages of ZIKV: the African and the Asian lineage [4]. It is estimated that the Asian lineage entered the Americas in 2013, where the ZIKV genome has experienced several mutations. The functional implications of these genomic changes are an active topic of study [13,14], while the rapid geographic dispersion was attributed to the ubiquitous presence of its vector: the mosquitoes of the genus *Aedes* [15,16]. Moreover, the finding that ZIKV is also sexually transmitted not only expanded the transmission channel to hosts [17,18] but also broadened the geographic reach to nontropical countries.

**Fig 1:**
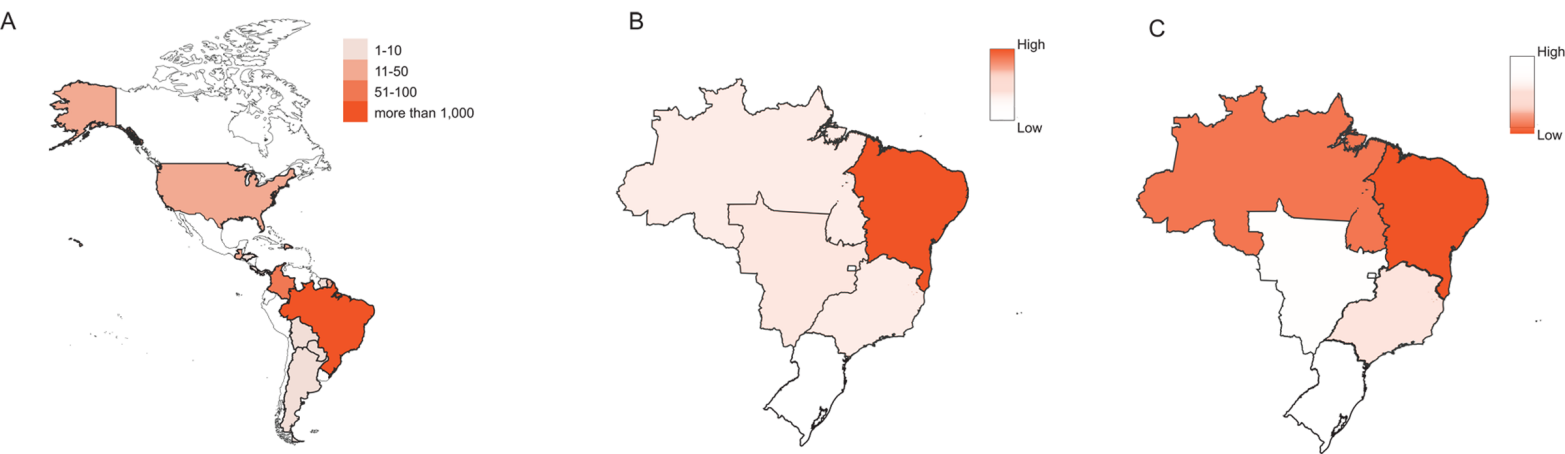
Geographical distribution of CZS. (A) Countries that have reported microcephaly and CNS malformation cases potentially associated with ZIKV infection until the end of 2016 according to WHO. Colors indicate the number of cases reported. (B) In Brazil (2015–2016), the prevalence of infection-related microcephaly varies according to the region. Here, the values for the rate between cases of ZIKV-related microcephaly and cases of ZIKV infection during pregnancy is displayed. Data was obtained from [19]. In the Northeast region, the amount of cases was relatively high (approximately 1,300 ZIKV-related microcephaly per 10,000 infected pregnants), while the Southeast region, for instance, showed lower prevalence (approximately 145 ZIKV-related microcephaly per 10,000 infected pregnants). (C) Distribution of average income per capita in each region of Brazil as informed by [37] for 2015. The lowest values correspond to the Northeast region. CNS, central nervous system; CZS, Congenital Zika Syndrome; ZIKV, Zika virus.

By the end of 2016, ZIKV infection had been reported in approximately 58 countries and across all the main continents; a total of 23 countries reported cases of microcephaly and other congenital anomalies potentially caused by ZIKV infection. Notably, 95% of all CZS cases in 2015 and 2016 were reported in Brazil, of which more than 75% were from the Northeast region of Brazil (Fig 1) [19]. In other words, although ZIKV outbreaks were registered in similar proportions across many geographic areas, informed cases of CZS have been clustered, indicating an asymmetric distribution among human populations [19,20]. This peculiarity opened several questions: why does the symptom distribution of such a widely spread virus vary so significantly by region and apparently also over time? Are there significant cofactors that help to determine the observed pattern?

## ZIKV evolution

One possible explanation for the sudden emergence of CZS is the proposition that the ZIKV genome changed immediately before or upon entering the Americas. A recent work has assessed this hypothesis. Yuan and colleagues [12] compared the neurovirulence of an ancestral Asian strain with a contemporary one isolated in the Americas. They found that contemporary Americas strains produce stronger effects on mouse brain development, both when infected prenatally and in newborns, than ancestral ones. The Americas ZIKV replicates at higher rates in the brain and disturbs cell proliferation and differentiation much more than the ancestral Asian ZIKV. According to their analyses, a single mutation, which occurred immediately before the outbreak in French Polynesia, is responsible for the differential neurovirulent effect [12]. This mutation largely coincided with the first reports linking ZIKV to neurological abnormalities, such as the Guillain–Barré syndrome, in 2013. The specifics with respect to how exactly ZIKV lineages differentiate themselves regarding their virulence and their impact on neural development is still under debate.

Evolutionary changes in ZIKV genome that facilitated the rapid geographic dispersion from Asia to the Americas and subsequently within the Americas should also be addressed. Some reports have stated that there were viral adaptations that enhanced the infectivity, both in human and mosquitoes [21–23]. These changes may explain the magnitude of the most recent Asian and Americas outbreaks, which are unprecedented in ZIKV history. In fact, it could be argued that the neurotropic effects of ZIKV were neglected prior to recent outbreaks, since former African and Asian epidemics infected fewer people in absolute terms, thus limiting visibility on patterns of congenital malformations substantially. Worth noting is the fact that infection with ZIKV provides immunity against posterior inoculation [24], and thus the impact of this virus now in the Americas could be related to large immunologically naive populations not previously exposed.

Altogether, viral genetic evidence of ZIKV’s evolution provides possible explanations on why CZS has been observed now but not in prior outbreaks outside the Americas. However, the scientific community remains unable to explain the high concentration of CZS in Brazil, especially the high concentration in its Northeast region. As such, focus has shifted beyond the virus itself to the examination of potential cofactors.

## Genetic and environmental cofactors

The impact of infectious diseases on human populations is variable and subject to population- and environmental-specific factors. Both of which potentially support the observed geographic clustering of CZS in Brazil.

Since immunodeficiency is at least in part inherited, a population’s genetic background modulates infection susceptibility. Regarding viral diseases, there are many examples in which the outcome of an infection depends on the genetic composition of the host. Susceptibility to human papillomaviruses (HPV) was partially understood when a genome linkage study showed that recessive mutations in two specific genes (*EVER1* and *EVER2*) were associated with the occurrence of cases of Epidermodysplasia verruciformis, a disease in which patients showed severe symptoms provoked by HPV and developed skin cancer [25]. In other words, people who have these mutations are more sensitive to HPV infection and consequently develop this rare disease. Another example is influenza virus, which manifests itself as a flu in around 10%–25% of the world’s population every year. The consequences of influenza infections vary from asymptomatic cases to death. Quantitative genetic studies on family pedigrees have shown that death caused by influenza is recurrent among relatives, suggesting that there is heritable predisposition to produce an insufficient immune response to this virus [26].

Animal models that have been used to experimentally reproduce physiopathological conditions related to ZIKV support the idea that the genetic background is relevant to infection. For example, in vivo experiments have demonstrated that adult wild-type mice, in contrast to type I IFN (Ifnar1) deficient mice, do not transfer ZIKV via the placenta efficiently. Approaches in which Ifnar1, a pathway response against viral infection [27,28], is disrupted, either through the administration of a blocking antibody [29–31] or the use of Ifnar1 knocked-out mice [28,30,32,33], are a suitable CZS model. In general, blocking Ifnar1 pathway before ZIKV infection leads to an increased viral load and a more compromised phenotype [28,30]. As such, genetics, as observed in mouse models, could be shaping the ZIKV effects in human populations.

**Fig 2:**
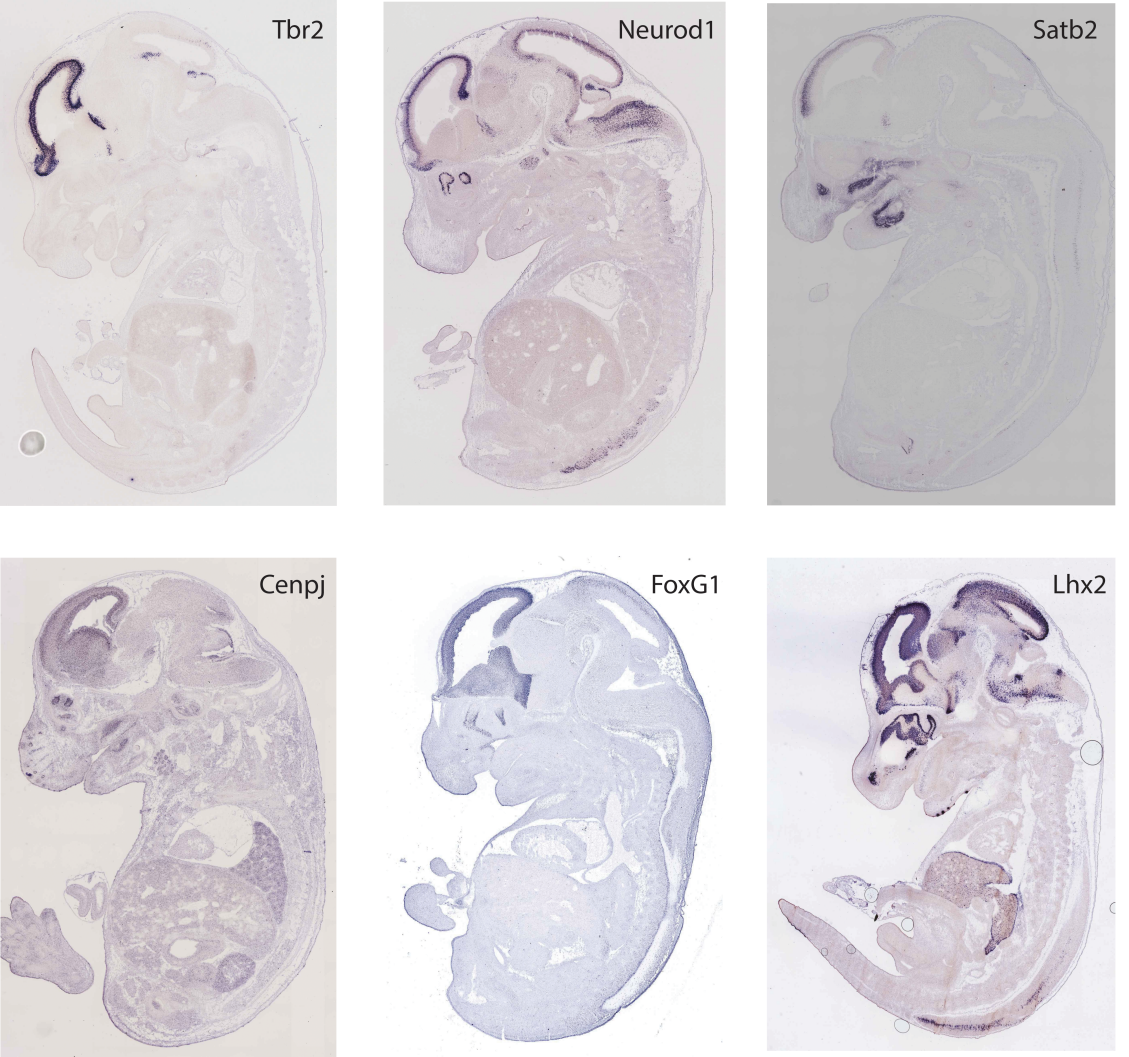
Gene expression is depicted using in situ hybridization in mouse samples of embryonic day (E15). Pictures were obtained from the open site GenePaint (*http*:*//www*.*GenePaint*.*org*).

Recently, the first systematic study of twins with CZS shed some light on the role of genetics [34]. According to its results, monozygotic twins, who were exposed to ZIKV during pregnancy, are both more prone to display CZS (concordant) than the pair of dizygotic twins, who are more likely to be discordant (one of the pair affected and the other one not). When analyzing gene expression of neural progenitor cells of discordant dizygotic twins, the study found that cells derived from those babies affected by CZS displayed a differential signature in neural development genes. This could explain, at least in part, the susceptibility of these cases to ZIKV infection [34]. Along these lines, analyses on differential gene expression could give important clues to formulate hypotheses regarding the role of genetics in CZS. In Table 2, we provide a summary of genes that have been found to be deregulated upon ZIKV infection. Interestingly, most of these genes identified as down-regulated after ZIKV infection have a robust expression in the embryonic neurogenic regions (Fig 2). However, it remains unanswered whether the ability of a pregnant woman to unfold a robust immune reaction to ZIKV infection and to prevent vertical transmission is explained by genetic background.

**Table 2:** Deregulated genes from representative biological processes upon ZIKV infection, verified in vitro and in vivo studies.

Biological Process	Genes	Expression	References
Antiviral response	*Mx1*, *Isg15*, *Off7*, *Oas2*, *Ifah1*, *Ddx58*, *Tlr2*, *Ddx6*, *eIF3c*, *DGCR8*, *IL17Ra*	Up-regulated	[35–37]
Potential ZIKV entry receptors	*Axl*	Up-regulated	[35]
Cell cycle	*Casc5*, *Rbbp8*, *Cdk6*, *Ccne2*	Down-regulated	[35–37]
Neural development	*Mcph1*, *Tbr2*, *Aspm*, *NeuroD1*, *Satb2*, *Cenpj*, *FoxG1*, *Lhx2*	Down-regulated	[34–37]

**Abbreviation:** ZIKV, Zika virus.

In addition to genetic background, environmental context can be a factor that impacts the immune status and response to ZIKV infection. In human populations, environmental factors that impact immune status are strongly related to socioeconomic position. Housing conditions, malnutrition, and coinfection susceptibility amongst other variables differ by region as well as by population and may contribute to differing responses to ZIKV infection.

As previously stated, the most affected area of Brazil regarding occurrences of CZS is the Northeast. Socioeconomic indicators reveal that this region has the lowest per capita average income in Brazil—amounting to around US$240 per month versus around US$450 in the Central-West [38] (Fig 1C). The dispersion is also reflected in more comprehensive parameters of social development, such as the Human Development Index (HDI), of which municipalities of the Northeast region have amongst the lowest scores in the country [39].

Epidemiological surveys tie demographic parameters to the prevalence of ZIKV congenital syndrome CZS [20,40]. When analyzing this relationship for Recife (Pernambuco), a city that was severely affected, it was found that cases of reported microcephaly in 2015 and 2016 were largely concentrated in areas with more impoverished living conditions [20]. In general, communities with lower socioeconomic status have more degraded housing, which facilitates ZIKV transmission by enabling mosquito’s easier reproduction and human access. In fact, dengue virus (DENV) prevalence in the same area of Brazil has been found to be strongly related to living conditions [41]. Although this ecological vector-based hypothesis provides a fertile area for further study and could describe a potential increase in infection cases, it may fall short of explaining why geographic regions characterized by similar levels of ZIKV infection and pregnancy are impacted differently by CZS. Two other hypotheses, which associated the CZS epidemic with the use of the larvicide pyriproxyfen or vaccine administration during pregnancy (Tdap) were dismissed by a recent case-control study in Recife [40].

Coinfections with other flaviviruses transmitted by the same vector (*Aedes* mosquito) could play a role in the immune response to ZIKV infection. DENV, for example, is endemic in South America, and its seroprevalence is found in more than 90% of the population within CSZ cluster regions [42]. It is known that after initial infection with DENV, posterior exposures to other DENV serotypes could result in life-threatening complications due to the phenomenon known as antibody-dependent enhancement. This describes a process in which antibodies generated during the first infection bind to the new virus but fail to efficiently neutralize it. Instead, the phenomenon facilitates virus entry to target cells.

Since ZIKV is structurally close to DENV, several studies have examined their cross-reactivity [43,44]. The studies demonstrated that antibodies generated after initial DENV infection bind with high levels of affinity to ZIKV and significantly enhance pathogenesis but ultimately fail to counteract the virus. The mechanism for this viral enhancement is thought to be mediated by immunoglobulin G engagement of Fc gamma receptors (FcyR), in agreement with other antibody-dependent enhancement examples [45].

Nutrition is an additional cofactor tied to both socioeconomic and demographic conditions, which is positively correlated with income as well as development levels and has been demonstrated to interfere with immunity. Malnutrition due to the insufficient intake of nutrients leads to impairment of immune function for several innate and adaptive pathways [46–49]. Further studies may explore the link between these specific cofactors and cases of birth defects produced after ZIKV infection.

## Missing gaps

Several aspects of ZIKV expansion and CZS prevalence can be obscured by confounding factors. First, the estimated number of infected patients is probably under-reported since a large proportion of infected individuals remain asymptomatic or with very mild and unspecific symptoms that could even be unnoticed. In fact, it is feasible that in previous ZIKV outbreaks, the relation between the infection and neurological diseases remained obscured by this underestimation of ZIKV infected cases. Second, the diagnosis for microcephaly is complicated because determination should be made based on the measure of head circumference with reference to age, sex, and body size. The latter point could be a problem since one of the features described in many cases of CZS is intrauterine growth restriction, which leads to a general reduction in body size/weight. Intrauterine growth restriction (IUGR) is a condition that is more frequent in vulnerable sectors of the society and in developing nations. It is broadly known that in cases of IUGR, brain size is relatively spared because its growth is preserved at expense of other tissues and organs [50]. Since the definition of microcephaly depends on body size, those children with lower body weight are expected to have even lower head circumference to be considered as a case of microcephaly. In this sense, many of those microcephalic cases found in Northeast Brazil during ZIKV outbreaks would be more extreme and probably more “noticeable.” This aspect could be considered one of the factors that contribute to overestimate CZS in lower socioeconomic classes. However, higher social classes have more access to private health services and more personalized care. Diagnosis of microcephaly would be accurate and careful in most of the cases in which body weight is normal. Therefore, the definition of microcephaly in CZS can be biased by these methodological and socioeconomic aspects as well. Further, immunological tests are still subjected to cross-reaction with other flaviviruses, interfering with an accurate diagnosis of viral infection. All these points complicate an estimation of the number of infected people in epidemiological studies.

Another important point that could bias epidemiological studies on CZS distribution is the differential access to reproductive health [51–53]. According to recent reports, after the massive publicity on the potential risks of congenital infection, Brazil observed a decline in the number of births. Since the number of fetal deaths did not change, this shift could be caused by a combination of postponement and pregnancy interruption [51]. By early 2016, health authorities from different Latin American countries recommended to postpone pregnancies to reduce the potential risk of CZS. However, in this region, more than half of pregnancies are unintended [54], revealing that this kind of recommendation likely only had a limited impact as a public health measure. Safe methods of contraception and abortion are not equally accessible for different socioeconomic cohorts. Regarding abortion, as it is an illegal practice in most Latin American countries, there is no reliable information on the actual number of cases. Further, abortion as well as pregnancy interruption for economic reasons is still a culturally stigmatized action [55]. Nevertheless, it has been reported that requests for information on abortion through Women on Web, an organization that provides access to mifepristone and misoprostol (two abortion medications), have significantly increased in those Latin American countries with large number of autochthonous ZIKV cases. In Brazil in particular, requests between November 2015 and March 2016 increased by 108% [56].

Finally, a recent experimental work on mice has demonstrated that the route of inoculation influences the rate of infection as well as the viral load found in the developing embryos. According to this study, sexual transmission results in more cases of infected embryos, reflecting that this route leads to more frequent vertical infection [57]. We can thus speculate that in social sectors with fragile reproductive health and prophylaxis, this route of infection would have a disproportionate impact. More population-based studies are necessary to properly test the hypothesis that there are differences in the prevalence of the infection route along populations and how this influences the distribution of CZS cases.

## An interdisciplinary challenge

The recent ZIKV outbreak created a long-term burden for affected nations with social and economic consequences that still cannot be fully quantified. The global biomedical research reaction to understand CZS, however, was remarkable. From these coordinated efforts, the necessity to strengthen and support epidemiological surveillance, which can not only help direct prevention but also provide information of scientific value for the future, became evident. ZIKV infection numbers dropped dramatically, and CZS patients born in 2017 are almost inexistent in South America [19]. A plausible explanation for this pattern may be the immunization of the vast majority of the at-risk population [23]. However, specific serological analyses remain necessary to confirm this.

In conclusion, ZIKV provides an example of how important constant dialogue between experimental, epidemiological, and viral vigilance work is. After ZIKV emerged as a medical concern at the end of 2015, answers to many questions came from the collaborative work of virologists, immunologists, epidemiologists, geneticists, neuroscientists, and developmental biologists, among others. To better understand why CZS is nonhomogeneously distributed among human populations, more interdisciplinary studies are needed. Future advances in understanding the role of cofactors in CZS should result from these interdisciplinary efforts.

## Abbreviations

CNS: central nervous system
CZS: Congenital Zika Syndrome
DENV: dengue virus
HDI: Human Development Index
HPV: human papillomavirus
IUGR: intrauterine growth restriction
STORCH: Syphilis, Toxoplasmosis, others, Rubella, Cytomegalovirus, and Herpes virus
ZIKV: Zika virus
